# Masterbatch of Chitosan Nanowhiskers for Preparation of Nylon 6,10 Nanocomposite by Melt Blending

**DOI:** 10.3390/polym14245488

**Published:** 2022-12-15

**Authors:** Se Bin Jin, Lam Tan Hao, Sung Yeon Hwang, Dongyeop X. Oh, Jun Mo Koo, Hyeonyeol Jeon, Sung Bae Park, Jeyoung Park

**Affiliations:** 1 Department of Chemical and Biomolecular Engineering, Sogang University, Seoul 04107, Republic of Korea; 2 Research Center for Bio-Based Chemistry, Korea Research Institute of Chemical Technology (KRICT), Ulsan 44429, Republic of Korea; 3 Department of Plant & Environmental New Resources, Graduate School of Biotechnology, Kyung Hee University, Namyangju-si 17104, Republic of Korea; 4 Department of Organic Materials Engineering, Chungnam National University, Daejeon 34134, Republic of Korea

**Keywords:** nylon 6,10, chitosan nanowhiskers, nanocomposite masterbatch, melt blending

## Abstract

Composite materials have been extensively studied to optimize properties such as lightness and strength, which are the advantages of plastics. We prepared a highly concentrated (30 wt %) nylon/chitosan nanowhisker (CSW) masterbatch by blending nylon 6,10 and CSW by solvent casting to achieve high dispersion efficiency while considering an industrial setting. Subsequently, 0.3 wt % nylon/CSW nanocomposites were prepared with a large quantity of nylon 6,10 via melt blending. During preparation, the materials were stirred in the presence of formic acid at different times to investigate the effect of stirring time on the structure of the CSW and the physical properties of the composite. The formation of nanocomposites by the interactions between nylon and CSW was confirmed by observing the change in hydrogen bonding using FT-IR spectroscopy and the rise in melting temperature and melting enthalpy through differential scanning calorimetry. The results demonstrated increases in complex viscosity and shear thinning. The rheological properties of the composites changed due to interactions between CSW and nylon, as indicated by the loss factor. The mechanical properties produced by the nanocomposite stirred for 1.5 h were superior, suggesting that formic acid caused minimal structural damage, thus verifying the suitability of the stirring condition.

## 1. Introduction

Chitosan is produced by the deacetylation of chitin, abundant in the exoskeleton of crustaceans and arthropods and the cell walls of higher plants such as fungi and algae. It is also the most abundant natural resource after cellulose [1,2,3]. Chitosan nanowhiskers (CSW) can be prepared from chitin crystals with a hierarchical structure by chemical treatment with a top-down approach [4,5,6,7,8,9,10]. Amino groups of the material become positively charged due to protonation in aqueous acid solution at low pH, and thus, dispersion is promoted by electrostatic repulsion [11]. When the net positive charge of chitosan increases due to protonation, polymer composites can be formed by ionic interaction with water-soluble negatively charged polymers [12,13,14,15,16,17,18,19]. In addition, nano-sized chitosan is already widely known and studied and is broadly used in medicine, polymers, textiles, and electrical engineering. Studies have been reported on tumor removal [20] and specific drug delivery, exploiting the abundant positively charged regions of chitosan and electrostatic attraction and repulsion [20,21,22], medical [23,24], and woodworking adhesives [25], using van der Waals force and salt bridges, coating films [26] that block the penetration of moisture and gas using the electrostatic attraction of chitosan and cellulose, flame-retardant gas barrier coatings that rely on electrostatic attraction to clay [27], and improved antibacterial performance of biodegradable mask filters through positively charged chitosan coating [28].

Nylon, a representative synthetic fiber, has excellent mechanical strength in the form of abrasion resistance, tensile strength, elasticity, dyeability, and chemical resistance. Due to these advantages, the demand for nylon by textiles and other industries has increased; thus, related research has been conducted [29,30,31,32]. In addition, research has been conducted on applying nylon to engineering nanocomposites by mixing with nanofillers such as graphene [33], cellulose nanocrystals [13,34], and silica nanoparticles [35].

Inorganic-based composites have been widely used to reinforce the insufficient physical properties of existing plastic materials [36,37,38,39]. Inorganic fillers can make polymers more rigid, flexible, and elastic while being present in nano or micro sizes in the matrix. However, because the interfacial bonding force between the inorganic fillers and the organic polymers is weak, it is difficult to achieve the desired effect relative to the input. In addition, its industrial use is prohibited due to health and environmental concerns. To solve the problem, we previously investigated all-organic nanocomposites and finally achieved excellent physical properties with only a small input [13,40,41]. However, there are limitations when applying the same process to already manufactured plastic as the nanofibers are dispersed in the monomer at the beginning of the polymerization process.

In this study, nylon/CSW nanocomposites based on nylon 6,10, with good mechanical properties and chemical resistance as an elastomer, and CSW, synthesized by deacetylation of the natural biomaterial chitin, were prepared by melt blending [42,43]. A nylon/CSW masterbatch was prepared in advance to promote the desired reactions between the polymer matrix and the nanofillers. In addition, samples were prepared using different stirring times to investigate the effect of exposure to formic acid on the structure of CSW during preparation and the change in physical properties.

## 2. Materials and Methods

### 2.1. Reagents

Commercial nylon 6,10 (HS22) was purchased from VESTAMID^®^, and formic acid (96%), sodium hydroxide (NaOH, 97%), hydrochloric acid (HCl, 37%), and shrimp shell α-chitin were purchased from Sigma-Aldrich. All reagents were used as received without further purification.

### 2.2. Synthesis of CSW

Before synthesizing CSW, chitin nanowhiskers (CNWs) were synthesized by the hydrolysis of shrimp shell α-chitin [10,13,44,45]. Bulk chitin powder (10 g) was placed in 3 M aqueous hydrochloric acid solution (250 mL). After the temperature was slowly increased to 120 °C, the reaction was carried out under nitrogen for 3 h. The prepared Chitin/HCl suspension was centrifuged at 2000 rpm for 20 min. The precipitate was washed three times by resuspension in water. The suspension prepared with washed chitin was sonicated for 10 min, and aqueous sodium hydroxide solution was added to the suspension to adjust the pH to 7–8. Then, the neutralized chitin suspension was transferred to a membrane tube and dialyzed in deionized (DI) water for 7 d to obtain CNW.

CSW was synthesized from CNW by deacetylation [10,13,46,47]. 250 mL of the suspension prepared with the synthesized CNW was transferred to a round-bottom flask, and purged with nitrogen; further, sodium hydroxide aqueous solution (30 wt %) was added to achieve a volume of 500 mL. The reaction was carried out at 80 °C for 6 h, and the samples were cooled at 25 °C and stirred for 24 h. Subsequently, the samples were centrifuged at 2000 rpm for 20 min and washed three times as in the synthesis of CNW. After the washed precipitate was recovered, a suspension was prepared and sonicated for 10 min, and an aqueous hydrochloric acid solution was added to adjust the pH to 7–8. The neutralized suspension was placed in a membrane tube, dialyzed in DI water for 7 d, and transferred to a conical tube. After completely freezing for 1 d, CSW was obtained by freeze-drying for approximately 5 d (Figure S1).

### 2.3. Preparation of Nylon/CSW Masterbatch

Before synthesizing all-organic nanocomposites by melt blending, a highly concentrated nylon/CSW masterbatch was prepared to promote the interaction between the polymer matrix and the bio-based filler. The nylon/CSW masterbatch was prepared by solvent casting. Nylon 6,10 was completely dissolved by stirring in formic acid to make a 6.5 wt % nylon solution. CSW (30 wt % relative to the solid content) was added to the prepared nylon solution, and the trapped air bubbles were removed through ultrasonication for 30 min. The nylon solution was stirred at room temperature at different stirring times (5 min–24 h) to promote the complete dispersion of CSW in the nylon solution. The homogenized nylon/CSW solution was cast in a Petri dish, dried at room temperature for 1 d, and then completely dried in a vacuum oven at 100 °C to obtain a highly concentrated nylon/CSW masterbatch.

### 2.4. Preparation of Nylon/CSW Nanocomposites Using Melt Blending

Nylon/CSW nanocomposites were prepared using a compounder (HAAKE MiniCTW, Mini Lab 3, Waltham, MA, USA) by extruding the prepared nylon/CSW masterbatch and nylon. The compounder has co-rotating screws. The barrel temperature was set at 240 °C with a screw speed of 50 rpm, and the by-pass cycle time was set to 3 min. Nylon/CSW masterbatch (1 wt % relative to nylon) was added, blended, extruded, and pelletized using a pelletizer. The prepared nylon/CSW nanocomposites were dried in a vacuum oven at 100 °C for 1 d and stored after vacuum sealing. The filler content in the final nanocomposite was 0.3 wt %.

### 2.5. Instrumental Analysis

#### 2.5.1. Mechanical Properties

The synthesized nylon/CSW nanocomposite samples were prepared for the tensile strength test by hot pressing at 240 °C for 10 min using a hot-press. The samples were tested according to the ASTM D638 Type V standard, which involved cutting them into dumbbell shapes with lengths and widths of 63.50 mm and 3.18 mm, respectively. The mechanical properties of the specimens were measured using a universal testing machine (UTM, Model 5943, Instron, Norwood, MA, USA). All tests were conducted in a controlled atmosphere at 18.9 °C and relative humidity of 35% using a thermo-hygrostat. Before testing the mechanical properties, the specimen was dried in a vacuum oven at 100 °C to remove water.

#### 2.5.2. Thermal Properties

Differential scanning calorimetry (DSC, Q2000, TA Instruments, New Castle, DE, USA) was performed in a nitrogen atmosphere from 0°C to 280 °C at a heating and cooling rate of 10 °C min^−1^. Data obtained from the second heating cycle were referred to as T_m_.

#### 2.5.3. Rheological Properties

Measurements using a dynamic rheometer (Rheometer, Anton Paar MCR 301, ARES, Graz, Austria) were performed by making a pre-made nylon/CSW nanocomposite film into a circular film with a diameter of 25 mm and a thickness of 0.5 mm under 5% strain, a frequency range of 0.05∼500 Hz, and a gap size of 1 mm at 280 °C. Rheological properties such as complex viscosity, storage modulus (G′), and loss modulus (G″) were analyzed using a dynamic rheometer.

#### 2.5.4. Fourier Transform Infrared Spectroscopy (FT-IR)

Infrared spectra of nylon/CSW nanocomposites were obtained using a Nicolet™ FTIR spectrometer (iS50, Thermo Scientific, Waltham, MA, USA); analysis was performed in attenuated total reflection mode. The samples were converted into films by a hot press and dried completely in a vacuum oven before analysis. Absorbance spectra were acquired for all samples. Each spectrum was acquired with 256 scans in the 4000–400 cm^−1^ range. The FT-IR absorbance spectrum was corrected for baseline shifts. To compare the spectra features among different samples, the peak intensities of the three samples were normalized by setting the maximum point to 1 and the minimum point to 0 based on the CH_2_ stretching (2942 cm^−1^) because the CH_2_ does not take part in nylon 6,10 inter-chain interaction.

## 3. Results

### 3.1. Synthesis of Nylon/CSW Nanocomposites

**Figure 1:**
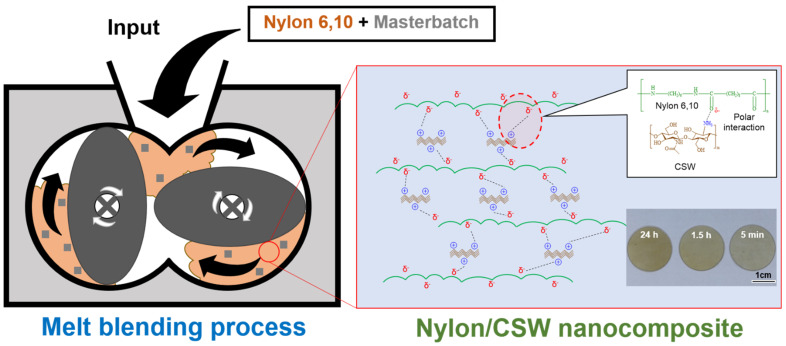
Preparation of nylon/chitosan nanowhisker (CSW) nanocomposite by melt blending.

Chitosan is produced by deacetylation from chitin, which is abundant in the exoskeleton of crustaceans and arthropods and the cell walls of higher plants. It is also the most abundant natural resource after cellulose. CSW prepared as described in Section 2 has an internal crystal structure. In addition, chitosan can become positively charged when the amino groups on its surface are protonated under acidic conditions (pH 7 or lower). The resulting electrostatic repulsion promotes the stable dispersion of the chitosan throughout aqueous media. Moreover, favorable interactions between the abundant positive charges on the chitosan surface and the polar amide groups of nylon can occur. Therefore, we prepared a polymer complex by exploiting the polar interactions between CSW, positively charged due to protonation by formic acid, and the amide groups of nylon (Figure 1). However, prolonged exposure of the chitosan material to acid can damage the chitosan nanostructure. Therefore, the changes in the mechanical strength and properties of the polymer composite were investigated after different exposure times, the longest of which replicated extreme conditions.

**Figure 2:**
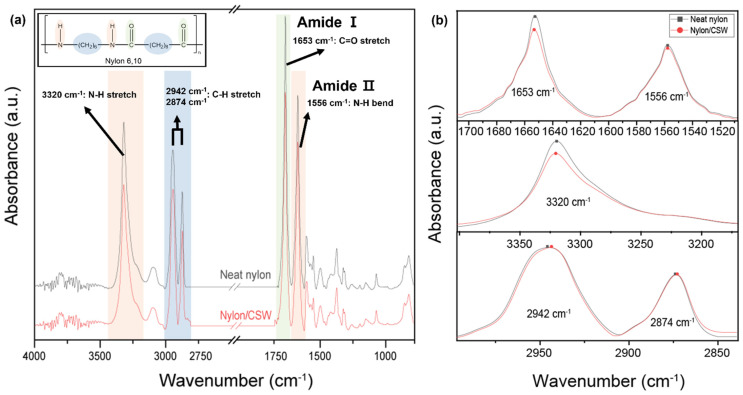
FTIR spectra of neat nylon and nylon/CSW nanocomposite (sample stirred for 1.5 h). (**a**) Full-range spectrum (600–4000 cm^−1^) and (**b**) Enlarged view of amide I (1653 cm^−1^, C=O stretch) and amide II (1556 cm^−1^, N-H bend) bonds, N-H stretch (3320 cm^−1^), and methylene groups (2942 and 2874 cm^−1^, C-H stretch).

The successful mixing of nylon and CSW was confirmed by comparing the major infrared spectroscopic peaks for pure nylon and the nylon/CSW nanocomposites (Figure 2) [48]. Figure 2a shows the positions and descriptions of each major peak in the 500–4000 cm^−1^ range. The peak intensities for the nylon/CSW nanocomposite at 3320 cm^−1^ for amide NH stretching and at 1556 cm^−1^ for amide NH bending were weaker than those of pure nylon due to the physical interaction between nylon and CSW (Figure 2b and Figure S2a). Similarly, the peak intensity at 1653 cm^−1^ for amide C=O stretching was lower due to interactions between nylon and CSW. In addition, the peaks at 2942 cm^−1^ and 2874 cm^−1^ corresponding to the methylene group of nylon had the same intensities in the spectra for the pure nylon and nylon/CSW nanocomposites. The intensity of hydrogen-bonded NH stretching (3020 cm^−1^) decreased and slightly shifted to a high wavenumber as the Lewis complexation free NH from hydrogen bonding. And, the signal of hydrogen-bonded C=O (1653 cm^−1^) exhibits a decrease in the intensity because Lewis acid complexation has a stronger electron-withdrawing effect on the C=O oxygen than hydrogen bonding (Figure S2b). Although our FTIR data did not show a significant peak shift, the change in peak intensity agrees well with the previous report [49]. The little change is probably due to the small loading level of CSWs. These results demonstrate that CSW formed composites by interactions with the amide group, not with the methylene chain of nylon.

**Figure 3:**
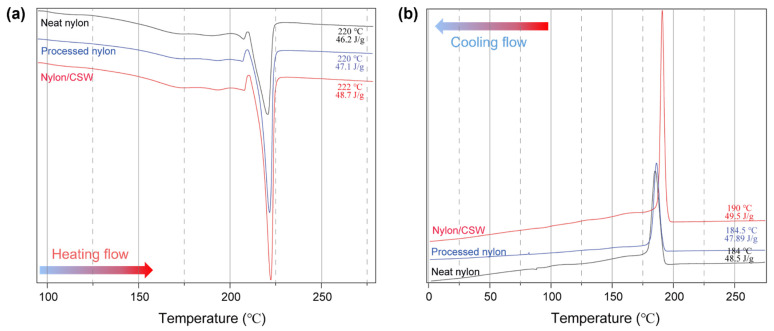
DSC thermograms of neat nylon, processed nylon, and nylon/CSW nanocomposite (sample stirred for 1.5 h). (**a**) Second melting temperature: T_m,_ and (**b**) first cooling crystallization temperature: T_c_.

The interactions between the nylon and CSW were confirmed using differential scanning calorimetry (Figure S3) [50]. The melting temperature of pure nylon and processed nylon are 220 °C, and the melting temperature of the nylon/CSW nanocomposite is 222 °C. In addition, the enthalpy of melting pure nylon of 46 J/g was slightly increased to that of 49 J/g in case of nanocomposite nylon with CSW (Figure 3a). Owing to the interactions between the CSW and nylon, a slightly higher heat capacity was required to melt the nylon/CSW nanocomposites than that for pure nylon. Next, the non-isothermal crystallization temperature obtained during cooling was 184°C for pure nylon and 190 °C for the nylon/CSW nanocomposite, approximately 6 °C higher for the nanocomposite (Figure 3b). Therefore, it is likely that CSW acts as a nucleating agent and increases the crystallization rate of polymers during the crystallization of nylon/CSW nanocomposites [51,52,53].

### 3.2. Mechanical Properties of Nylon/CSW Nanocomposites

**Figure 4:**
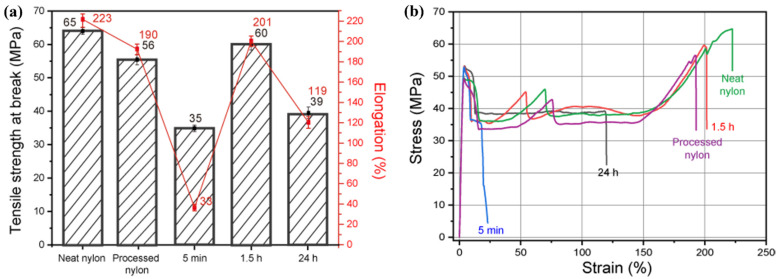
Mechanical properties resulting from various stirring and acid exposure times for the nylon/CSW nanocomposite. (**a**) Elongation (red line) and ultimate tensile strength (black column) and (**b**) stress–strain curve.

Figure 4 shows the mechanical properties resulting from different synthesis conditions for the nylon/CSW composites. Different stirring times (24 h, 1.5 h, and 5 min) were tested using three different samples in formic acid. Nylon without CSW was used as a control sample. Although the physical properties of the nylon/CSW nanocomposite were found to be generally inferior to those of nylon, heat-processed nylon decreased mechanical properties than the 1.5 h sample due to heat damage. In addition, the physical properties of the samples stirred for 24 h and 5 min demonstrated more deterioration than the samples stirred for 1.5 h. These results demonstrate that when CSW was exposed to formic acid for a long time, the reinforcing effect decreased as the nanostructure was damaged. However, when the stirring time was too short, aggregation of the CSWs occurred because the CSWs were not sufficiently dispersed in the solution. Therefore, cracks were induced during a tensile test after film production, and the elongation rapidly decreased.

**Figure 5:**
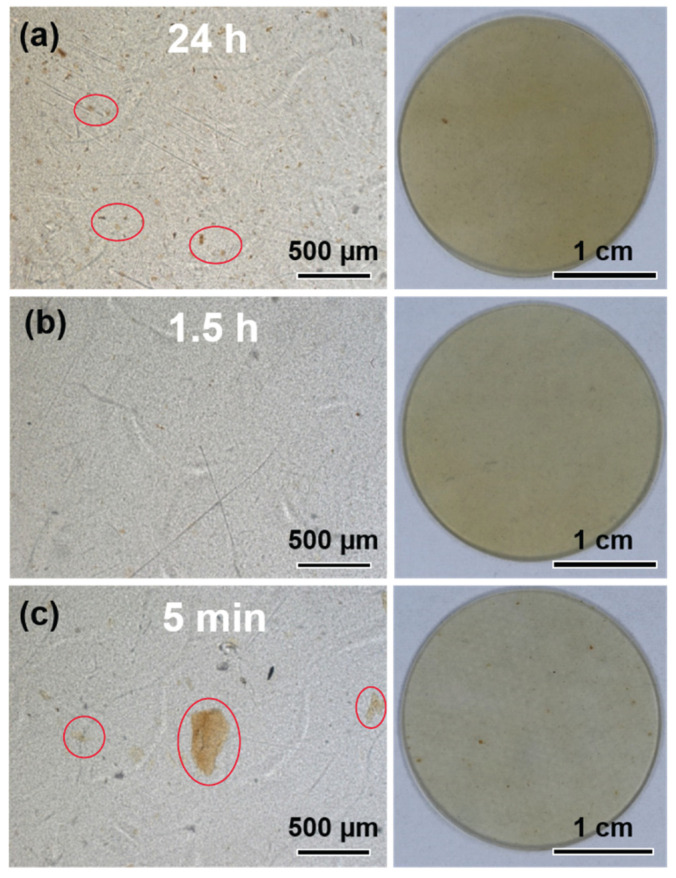
Optical microscopic and digital images of nylon/CSW nanocomposite (**a**) 24 h, (**b**) 1.5 h, and (**c**) 5 min.

The relationship between acid exposure time and structural damage of CSW was analyzed using an optical microscope. Surface images of the samples exposed to acid for different lengths of time during masterbatch preparation are shown in Figure 5a–c. In samples subjected to 24 h stirring and acid exposure time, the CSW was uniformly dispersed among the nylon due to the long stirring time [54,55,56]. However, the damaged chitosan nanostructure as the exposure time to acid increased, causing the aggregation of organic matter. Therefore, we reduced the stirring and acid exposure time to minimize the collapse of the structure and aggregation of CSW. After 1.5 h stirring and acid exposure time, the nanostructure was not damaged, and CSW aggregation did not occur (Figure 5b), due to the short acid exposure time. There was no damage to the chitosan nanostructure in the sample stirred for 5 min—the shortest stirring and acid exposure time. However, due to insufficient agitation, more aggregation was observed [57].

### 3.3. Rheological Properties of Nylon/CSW Nanocomposites

**Figure 6:**
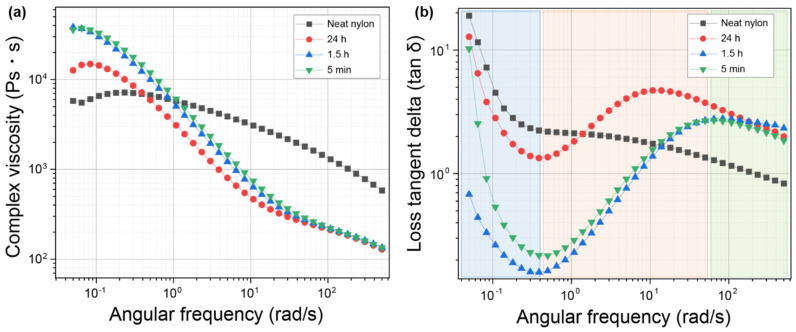
Rheological properties of nylon stirred with formic acid for different lengths of time to form a nylon/CSW nanocomposite. (**a**) Complex viscosities and (**b**) loss tangent delta (tanδ) of nylon and nylon/CSW nanocomposite subjected to different acid exposure times calculated using frequency sweep experiments.

The melt viscosity behavior of a linear polymer such as nylon can be divided into three stages according to the shear rate: the lower Newtonian stage (low shear rate), the pseudoplastic flow stage (medium shear rate), and the upper Newtonian stage (high shear rate). Figure 6a shows the relationship between the complex viscosity (η) and the angular frequency (rad/s) of pure nylon 6,10, and nylon/CSW nanocomposites. The angular frequency corresponds to the shear rate. In general, the complex viscosity in the low Newtonian region is called the zero shear viscosity. The zero shear viscosity is highly dependent on the molecular weight of the polymer and the influence of nanofillers. Higher zero shear viscosities were obtained for the nylon/CSW nanocomposites than for pure nylon because the movement of polymer melts was restricted due to the entanglement of CSW in the molecular chains of nylon. The composite samples stirred for 1.5 h and 5 min demonstrated relatively lower zero shear viscosity than the composite samples stirred for 24 h because the nanostructure of the sample stirred for 24 h was damaged [54,58]. In the pseudoplastic flow region, the polymer is oriented, and the viscosity decreases as the shear rate increases, according to the shear-thinning effect. The shear-thinning effect of the nylon/CSW nanocomposite was more significant than that of pure nylon because the interactions between CSW and nylon can promote the orientation of the polymer chains.

Prolonged stirring of CSWs in formic acid can lead to partial dissolution and de-polymerization. Thus, the CSWs molecules can split with less perfect crystal than the original CSWs and increase the number of molecules which may form a ‘polymer blend’ with nylon 6,10. The DSC curve of the 24 h sample (nylon/CSWs composite) is similar to those of other samples. The melting temperature and enthalpy of nylon 6,10 shifted to a higher temperature and increased in the 24 h sample compared to other composite samples which could lead to an increase in the nuclei due to the partial de-polymerization after the 24 h stirring process. The role of polysaccharide nanomaterials to act as a nucleating agent has been observed for other polyamide masterbatches produced via formic acid-blending and melt extrusion [59], and other polymer nanocomposite systems/masterbatches [60,61,62,63]. Moreover, the CSW masterbatch was added only 1% compared to nylon, and the final content of CSW was 0.3 wt % compared to nylon. Therefore, it is difficult to confirm the dramatic difference in DSC analysis.

Figure 6b shows the relationship between the loss tangent delta (tan δ = G′/G″) and the angular frequency for each nylon/CSW nanocomposite sample. The loss tangent delta is the value obtained by dividing storage modulus (G′; elastic part or stored energy) by the loss modulus (G″, viscous part or energy loss) and is predominantly used to identify the tendency of material viscoelasticity. Liquid properties become stronger as the loss tangent delta increases; conversely, solid properties become stronger as the loss tangent delta value decreases. The loss tangent delta of pure nylon gradually decreased as the shear rate increased, a common characteristic of fluids. The higher the deformation rate, the stronger the solid properties of the material, which is why water feels hard when diving in a pool. However, in the case of the nylon/CSW nanocomposites obtained by stirring for 5 min and 1.5 h, the loss tangent delta values rapidly decreased at the low shear viscosity stage. After the loss tangent delta values of the samples rapidly decreased, they began to gradually increase with the shear rate. The fluid flow was significantly hampered by the entanglement of the CSW with the polymer chain in the low shear rate region. In contrast, the nylon polymer chain was oriented, and the polymer chain and CSW were not tangled at the high shear rate stage.

## 4. Conclusions

Organic matter-based CSW was used to promote interactions between a nylon polymer matrix and the nanofillers. In addition, a nylon/CSW masterbatch was prepared in advance to achieve the high dispersibility required to apply this research in the industry. Samples were prepared by stirring for different acid exposure times to investigate the effect of formic acid on the structure of the CSW and its physical properties. The final nylon/CSW nanocomposites were prepared by melt blending using a compounder and analyzed using DSC, FT-IR, rheometry, and UTM. Compared to pure nylon, the nylon/CSW nanocomposite exhibited a higher melting enthalpy and crystallization temperature, possibly due to the interactions between CSW and nylon. An evaluation of the mechanical properties indicated that the physical properties deteriorated as the stirring time increased during nylon/CSW masterbatch production. In addition, with a short acid exposure time, sufficient protonation of the CSW did not occur, and thus the physical properties deteriorated. Based on our evaluation of dynamic rheological properties, the complex viscosity in the low angular frequency region of the composite was significantly higher than that of pure nylon. In addition, the complex viscosity increased significantly in the samples prepared using a short stirring time. The changes in the rheological properties of the composite due to the interactions between CSW and nylon were confirmed by examining the shear-thinning effect, which was more significant for the composite than for pure nylon, indicating that the composite has higher viscoelasticity.

## Data Availability

The data presented in this study are available on reasonable request from the corresponding author.

## Supplementary Materials

The following supporting information can be downloaded at: https://www.mdpi.com/article/10.3390/polym14245488/s1, Figure S1: SEM image of CSWs; Figure S2: Normalized FTIR spectra of neat nylon, nylon/CSW composite, and CSW; Figure S3: DSC thermograms of neat nylon, processed nylon, and nylon/CSW nanocomposite.
