# Supplementary material for: Masterbatch of Chitosan Nanowhiskers for Preparation of Nylon 6,10 Nanocomposite by Melt Blending
Source: Polymers (Basel). 2022 Dec 15;14(24):5488. doi: 10.3390/polym14245488 (PMC9783613; doi:10.3390/polym14245488)
Supplement: Supplementary file 1 [file polymers-14-05488-s001.zip › polymers-2021023-supplementary.pdf]

Supplementary Materials: Masterbatch of chitosan nanowhiskers for preparation of nylon 6,10 nanocomposite by melt blending

Se Bin Jin[1,2], Lam Tan Hao[2], Sung Yeon Hwang[2,3], Dongyeop X. Oh[2], Jun Mo Koo[2,4], Hyeonyeol Jeon[2], Sung Bae Park[2,*], Jeyoung Park[1,2,*]

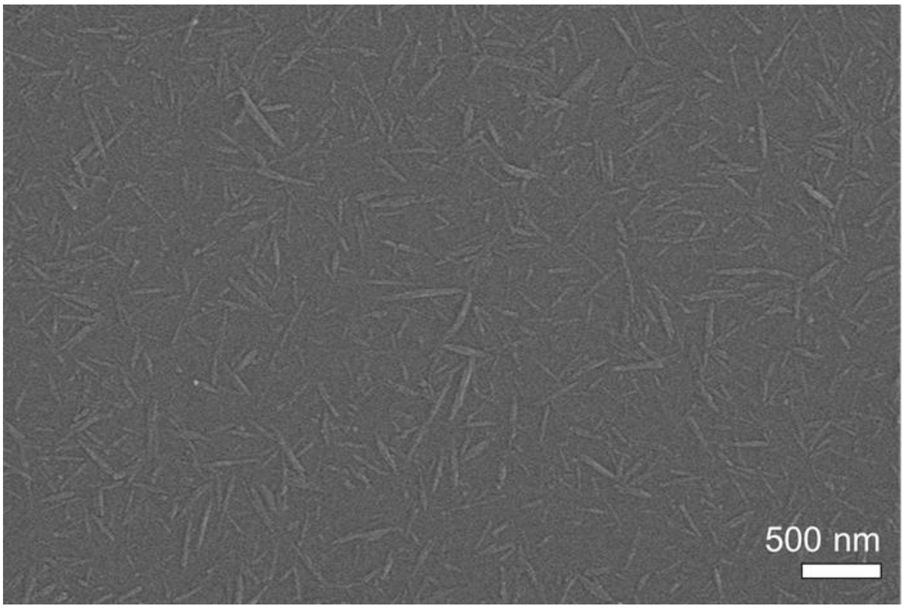

Figure S1. SEM image of CSWs.

Method: The morphology of CSWs was examined by field-emission scanning electron microscopy (SEM; Tescan MIRA3, Czech Republic). The sample was sputter-coated with a platinum to reduce the charge effects prior to measure with SEM (150 mA, 90 s).

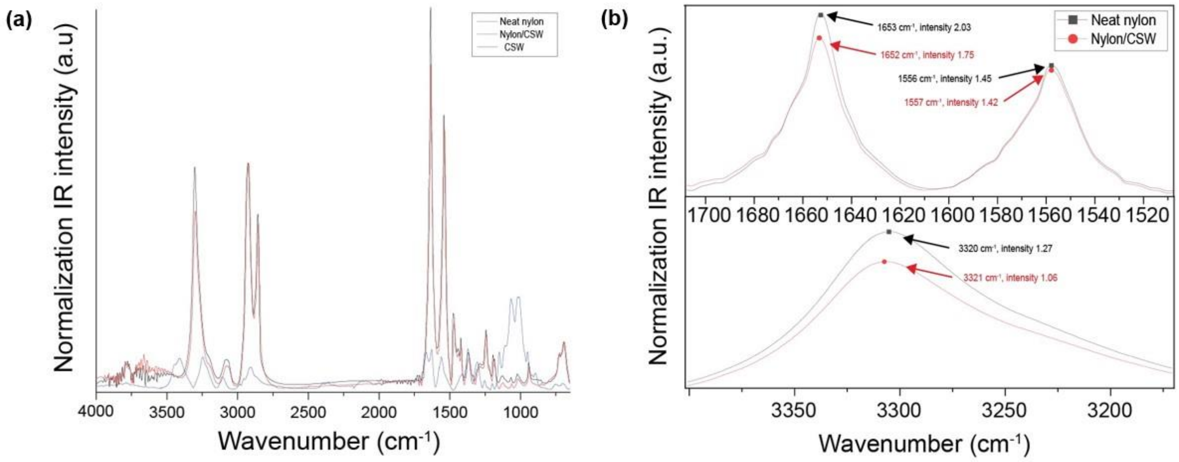

Figure S2. Normalized FTIR spectra of neat nylon, nylon/CSW composite (sample stirred for 1.5 h), and CSW. (a) Full-range spectrum (600–4000 cm^{-1}) and (b) Enlarged view of amide I (1653 cm^{-1}, C=O stretch), amide II (1556 cm^{-1}, N-H bend) bonds and N-H stretch (3320 cm^{-1}).

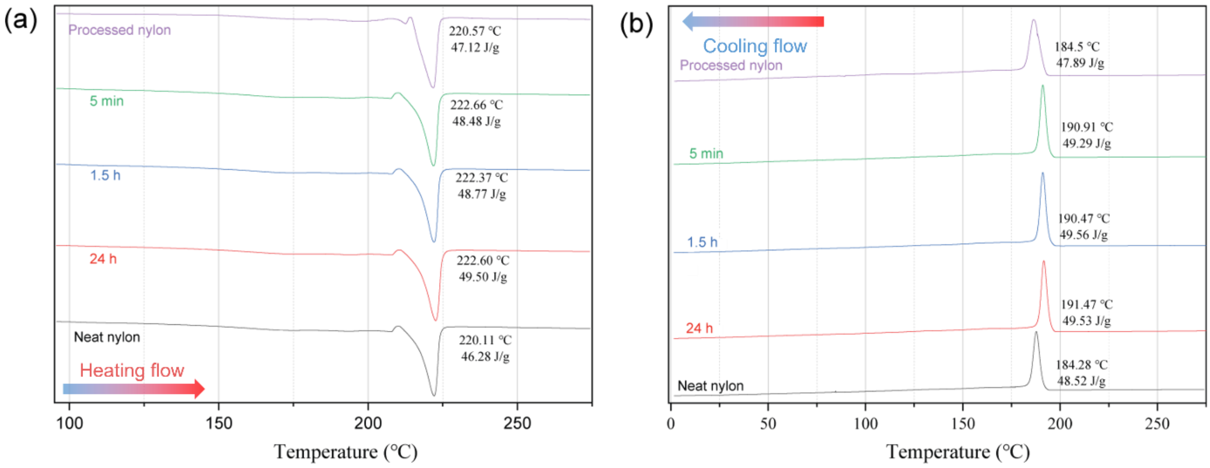

Figure S3. DSC thermograms of neat nylon, processed nylon, and nylon/CSW nanocomposite (sample stirred for 5 min, 1.5 h, and 24 h). (a) Second melting temperature: T_m, and (b) first heating crystallization temperature: T_c.
